# New insights into the characteristic skin microorganisms in different grades of acne and different acne sites

**DOI:** 10.3389/fmicb.2023.1167923

**Published:** 2023-04-27

**Authors:** Zitao Guo, Yuliang Yang, Qianjie Wu, Meng Liu, Leyuan Zhou, Liang Zhang, Dake Dong

**Affiliations:** ^1^National Engineering Research Center of Cereal Fermentation and Food Biomanufacturing, Jiangnan University, Wuxi, China; ^2^Jiangsu Provincial Research Center for Bioactive Product Processing Technology, Jiangnan University, Wuxi, China; ^3^Wuxi Medical College, Jiangnan University, Wuxi, China; ^4^Department of Radiation Oncology, Dushu Lake Hospital Affiliated to Soochow University, Suzhou, China; ^5^Department of Dermatology, Affiliated Hospital of Jiangnan University, Wuxi, China

**Keywords:** skin microbiota, acne, body site, *Pseudomonas*, *Ralstonia*

## Abstract

**Background:**

The increasing maturity of sequencing technology provides a convenient approach to studying the role of skin microorganisms in acne pathogenesis. However, there are still too few studies about the skin microbiota of Asian acne patients, especially a lack of detailed analysis of the characteristics of the skin microbiota in the different acne sites.

**Methods:**

In this study, a total of 34 college students were recruited and divided into the health, mild acne, and severe acne groups. The bacterial and fungal flora of samples were separately detected by 16S and 18S rRNA gene sequencing. The biomarkers of different acne grades and different acne sites [forehead, cheek, chin, torso (including chest and back)] were excavated.

**Results and Discussion:**

Our results indicated that there was no significant difference in species diversity between groups. The genera like *Propionibacterium, Staphylococcus, Corynebacterium*, and *Malassezia*, which have a relatively high abundance in the skin microbiota and were reported as the most acne-associated microbes, were no obvious differences between groups. On the contrary, the abundance of less reported Gram-negative bacteria (*Pseudomonas*, *Ralstonia*, *and Pseudidiomarina*) and *Candida* has a significant alteration. Compared with the health group and the mild group, in the severe group, the abundance of *Pseudomonas* and *Ralstonia* sharply reduced while that of *Pseudidiomarina* and *Candida* remarkably raised. Moreover, different acne sites have different numbers and types of biomarkers. Among the four acne sites, the cheek has the greatest number of biomarkers including *Pseudomonas*, *Ralstonia*, *Pseudidiomarina*, *Malassezia*, *Saccharomyces*, and *Candida*, while no biomarker was observed for the forehead. The network analysis indicated that there might be a competitive relationship between *Pseudomonas* and *Propionibacterium*. This study would provide a new insight and theoretical basis for precise and personalized acne microbial therapy.

## 1. Introduction

The skin is the largest organ in the human body, with a total mass of about 16% of the body weight (Human Microbiome Project Consortium, 2012). It is the first defense line to protect the body against pathogens. Theoretically, the skin environment is not conducive to the growth and reproduction of microorganisms, mainly due to the sweat secreted by the skin will acidify the skin and skin tissues or cells will release antibacterial ingredients such as antibacterial peptides. However, the hair follicles, sebaceous glands, sweat glands, and other appendages in the skin provide a variety of growth spaces for microorganisms and form a relatively stable skin microbiota (Sender et al., 2016). The skin is the second largest microecosystem of the human body after the gut. It has been reported that the number of adult skin microorganisms is about 40 trillion, accounting for 16% of the total number of human symbiotic microorganisms (Molly et al., 2009; Human Microbiome Project Consortium, 2012). For a long time, the awareness of people for skin microorganisms has been limited to a few culturable microorganisms, such as *Propionibacterium*, *Staphylococcus*, and *Malassezia* (Gao et al., 2007). With the rapid development of sequencing technology, researchers have a deeper understanding of the composition and function of skin microorganisms. In recent years, there were many studies have proved that skin microorganisms play a vital role in participating in the regulation of the skin’s physiological microenvironment, mediating inflammation and immune responses. It has a tight relationship with the occurrence and development of skin diseases (Chen et al., 2018; Schneider et al., 2023).

Acne is a common chronic inflammatory and hair follicle sebaceous gland disease in dermatology. It tends to occur on the cheek and forehead, followed by seborrheic sites such as the chest, back and shoulders. The clinical manifestations are mainly acne, papules, pustules, nodules, cysts, and scars and other skin lesions. Acne affects 9.4% of the global population and is the eighth most prevalent disease in the world (Kong et al., 2017). Epidemiological studies have shown that 80 ~ 90% of adolescents have suffered from acne, and the incidence of acne continues to rise with changes in lifestyle, environment, and climate. More serious, acne usually appears on the face, some patients may have different degrees of atrophic and/or hypertrophic scarring in the later stages of the disease, which has a great impact on the patient’s mood, daily activities, and interpersonal relationships (Hazarika and Archana, 2016). The main pathogenesis of acne is the homeostatic imbalance of the sebaceous gland, hyperkeratosis of the hair follicular epithelium, imbalance of microecology and inflammatory response (Yu et al., 2020). Although the specific mechanism of acne development is unknown, the involvement of microorganisms is one of the important mechanisms of acne pathogenesis. Currently, the wide application of high-throughput sequencing technologies such as metagenomic sequencing and amplicon sequencing has promoted the study of the role of skin microorganisms in the pathogenesis of acne. However, skin samples in most of these studies were mainly from western subjects (Human Microbiome Project Consortium, 2012; Leung et al., 2015). Given that the significant differences in diet and lifestyle of different racial groups will affect the composition of skin microbiota, there is a need to increase samples from populations in other regions.

In recent years, appreciation for investigating the skin microorganisms of acne patients in Asia has increased. The literature review indicated that the first study of the skin microbiota of Asian acne patients with different-grade (grading was determined according to the 2016 European S3 Acne Guideline) was reported by China institutions in 2019 (Li et al., 2019). The skin samples were taken from the cheek surface of the subjects. The 16S rRNA gene sequencing revealed that the skin microbiota of acne patients has a close relationship with the grade of acne and a significant difference was observed between grade 4 acne and grade 1–3 acne. Subsequently, in 2021, the first study to characterize the communities of bacterial and fungus in acne patients was reported by Korean institutions (Kim et al., 2021). The microbiome and mycobiome of the forehead and right cheek surface samples from the Korean female subjects were determined by 16S rRNA gene sequencing and internal transcribed spacer-1 (ITS1) region sequencing. They found that the ratio of *Cutibacterium* to *Staphylococcus* of the acne group is higher than that of the health group, while no difference was observed in the alpha and beta diversity of bacterial and fungi communities between groups. In the same year, two studies about the skin microorganisms of Chinese acne patients were reported (Shi et al., 2021; Xu et al., 2021). Contrary to the previous Korean team’s findings, a comparison of the microbial composition of the skin surface showed significant differences in microbiota diversity between the acne group and the health group (Shi et al., 2021). In another study, researchers paid more attention to the role of skin microorganisms within the pilosebaceous unit during the transformation of non-inflammatory to inflammatory acne lesions (Xu et al., 2021). They found that *Malassezia restricta* and *Staphylococcus epidermidis* might play the same important role as *Cutibacterium acnes* in the development of inflammatory lesions in acne patients due to they have the similar proliferation trends. Notably, the same findings were also reported in a recent study that detect the skin microorganisms of Japanese acne subjects (Akaza et al., 2022).

Although the above research has characterized the skin microorganisms’ communities in Asian acne patients, there are still too few studies relative to the large population. In addition, some shortages could be observed in these studies. For example, out of the three reports on skin microorganisms in Chinese acne patients, only one study analyzed the communities of fungi in skin samples (Li et al., 2019; Shi et al., 2021; Xu et al., 2021). Moreover, many studies focus on comparing the difference in the composition of skin microbiota between the acne patients and the healthy controls, there is a lack of detailed analysis about the characteristics of skin microbiota structure in the acne sampling sites. To overcome these disadvantages and provide targeted skin microorganisms to dermatologists for the treatment of acne, in this study, the skin microbiota structure of different acne grades and different sampling sites (forehead, cheek, chin, chest, and back) were investigated by 16S and 18S rRNA gene sequencing. The biomarkers were excavated and the functions of skin microbiota were predicted. This study would provide support for dermatologists to develop microbiological approaches for treating acne.

## 2. Materials and methods

### 2.1. Subject recruitment

All subjects were recruited from college students at Jiangnan University (Wuxi city, Jiangsu Province, China) from May to June 2022. The participant enrolled in this pilot was recruited by strictly obeying the exclusion criteria as follows: No oral antibiotics, hormones, tretinoin preparations, or immunosuppressants within 6 months; Not applied any topical drugs or functional cosmetics to the face within 1 month; Individuals without other skin lesions on the face and absence of systemic disease. Finally, a total of 34 participants were recruited. Among these, the subjects with mild and severe acne lesions, grade 1 and grade 4 according to the Pillsbury scoring method (Witkowski and Parish, 2004), were 20 and 6, respectively. Besides, 8 health subjects with no skin problems and who obeyed the above exclusion criteria were recruited as a control group. All participants gave written informed consent in accordance with the Declaration of Helsinki and this study was approved by the ethics committee of the Affiliated Hospital of Jiangnan University (approval no. LS2022046).

### 2.2. Sample collection

All participants were asked to wash their faces with a neutral facial cleanser, avoid re-washing and avoid other contamination for 24 h thereafter, and not use any skin care products, makeup, perfumes, and topical drugs before sampling. Acne skin surface microbiota located on the forehead, cheeks, chin, and back and chest of subjects in the acne groups were sampled, and the same skin sites of the healthy control group were also sampled. To avoid the influence of different operators on the sample detection, the same operator conducted the clinical observation and took the measurements. All sampling operations were carried out in a room with constant temperature and humidity (25°C, 40–50%). The sample collector wore sterile gloves and placed the 75% ethanol-disinfected hollow film (4 cm diameter) at the sample site (the detailed operation was shown in Supplementary Figure S1). Then, samplings were proceeded by using a saline-soaked sterile flocking swab to wipe the sample site 40 times (10 times each horizontally, vertically, left, and right obliquely). After that, each swab was collected in a sterilized centrifuge tube and stored at a − 80°C refrigerator within 30 min until extracting the genomic DNA.

### 2.3. DNA extraction and sequencing

The genomic DNA of each sample was extracted by using a commercial DNA kit (Nanjing Vazyme Biotech Co., Ltd., Nanjing, China) and following the manufacturer’s instructions. After the detection of extracted DNA purity and concentration using agarose gel electrophoresis, an appropriate amount of DNA was then taken in a centrifuge tube and diluted to 1 ng/μL with sterile water for subsequent sequencing. The primers 341F (5′-CCTAYGGGRBGCASCAG-3′) and 806R (5′-GGACTACNNGGGTATCTAAT-3′) were employed to amplify the V3-V4 regions of the 16S rRNA genes of all bacterial samples, while the primers 528F (5′-GCGGTAATTCCAGCTCCAA-3′) and 706R (5′-AATCCRAGAATTTCACCTCT-3′) were applied to amplify the V4 region of the 18S rRNA genes of all eukaryotic samples. The PCR reaction system (30 μL) was composed of a 15 μL mix of highly effective high-fidelity enzyme and buffer (Phusion^®^ High-Fidelity PCR Master Mix with GC Buffer, New England Biolabs, United States), 2 μL primers (the forward and the reverse was 1 μL, respectively), genomic DNA 10 μL, 3 μL double distilled water (ddH_2_O). The amplification steps were as follows: pre-denaturation at 98°C for 1 min; 30 cycles of 98°C for 10 s, 50°C for 30 s, 72°C for 30 s. Then, 72°C for 5 min for all samples. After purification, the final PCR products were used for library construction by employing the NEB Next^®^ Ultra DNA Library Prep Kit (New England Biolabs, United States). Once the library was qualified, the NovaSeq 6000 (Illumina, San Diego, CA, United States) was used for sequencing.

### 2.4. Bioinformatic analysis

The raw data of all samples were filtered, denoised, merged, and dechimeric to form ASVs with Qiime2’s DADA2 plugin. The taxonomy assignments proceeded based on the Greengenes (for 16S rRNA gene sequencing) and the UNITE databases (for 18S rRNA gene sequencing). The alpha diversity was represented by the index of Shannon. The beta diversity was displayed by principal coordinate analysis (PCoA) plots. The differences at the genus level of the skin microbiota between different groups were analyzed based on their relative abundance. The skin surface microbiota function was predicted based on the KEGG (Kyoto Encyclopedia of Genes and Genomes) database and MetCyc (Metabolic Pathways From all Domains of Life) by using the software PICRUSt. After the functional annotation, the Dunn’s test package of R language was used to statistically analyze the significant difference in the microbial community prediction function between groups.

### 2.5. Statistical analysis

The obtained experimental data were statistical analyzed by using SPSS 25.0 (SPSS Inc., Chicago, IL, United States) with following procedure. Firstly, the Gaussian distribution of data was checked by using the Shapiro–Wilk test. After that, the one-way ANOVA test was employed to analysis the Gaussian distribution data, and the LSD test and the Games-Howell test was applied to conduct multiple comparison when the data with or without equal variances assumed. Otherwise, the statistical difference of different groups was judged by the Kruskal-Wallis test. Unless otherwise indicated, the data are presented by mean ± standard deviation. The significant difference was accepted at *p* < 0.05, while *p* < 0.01 was supposed as a highly significant difference.

## 3. Results

### 3.1. Characteristics of study cohort

In this study, a total of 34 participations were recruited. In the health control and mild group, the ratio of females to males was 1:1. Among subjects with severe acne, there were 5 males and 1 female. The age of subjects ranged from 19 to 26, the average age of the health, mild, and severe group was 23.5 ± 1.9, 22.1 ± 2.3, and 20.8 ± 1.3, respectively. There was no difference in age between groups (*p* > 0.05, LSD test). The total number of samples in the health, mild, and severe groups was separately 20, 22, and 19. To compare the skin microbiota structure between different acne lesion sites, the samples were divided into four groups based on their sampling sites and the number of each sampling site was listed in Table 1.

**Table 1:** The basic information of subjects and samples.

	Health	Acne-mild	Acne-severe
Sex (F/M)	8 (4/4)	20 (10/10)	6 (1/5)
Age (years)	23.5 ± 1.9	22.1 ± 2.3	20.8 ± 1.3
Forehead	5	8	5
Cheek	6	4	7
Chin	6	5	3
Chest and back	3	5	4
Total	20	22	19

### 3.2. Species diversity of skin microbiota

**Figure 1:**
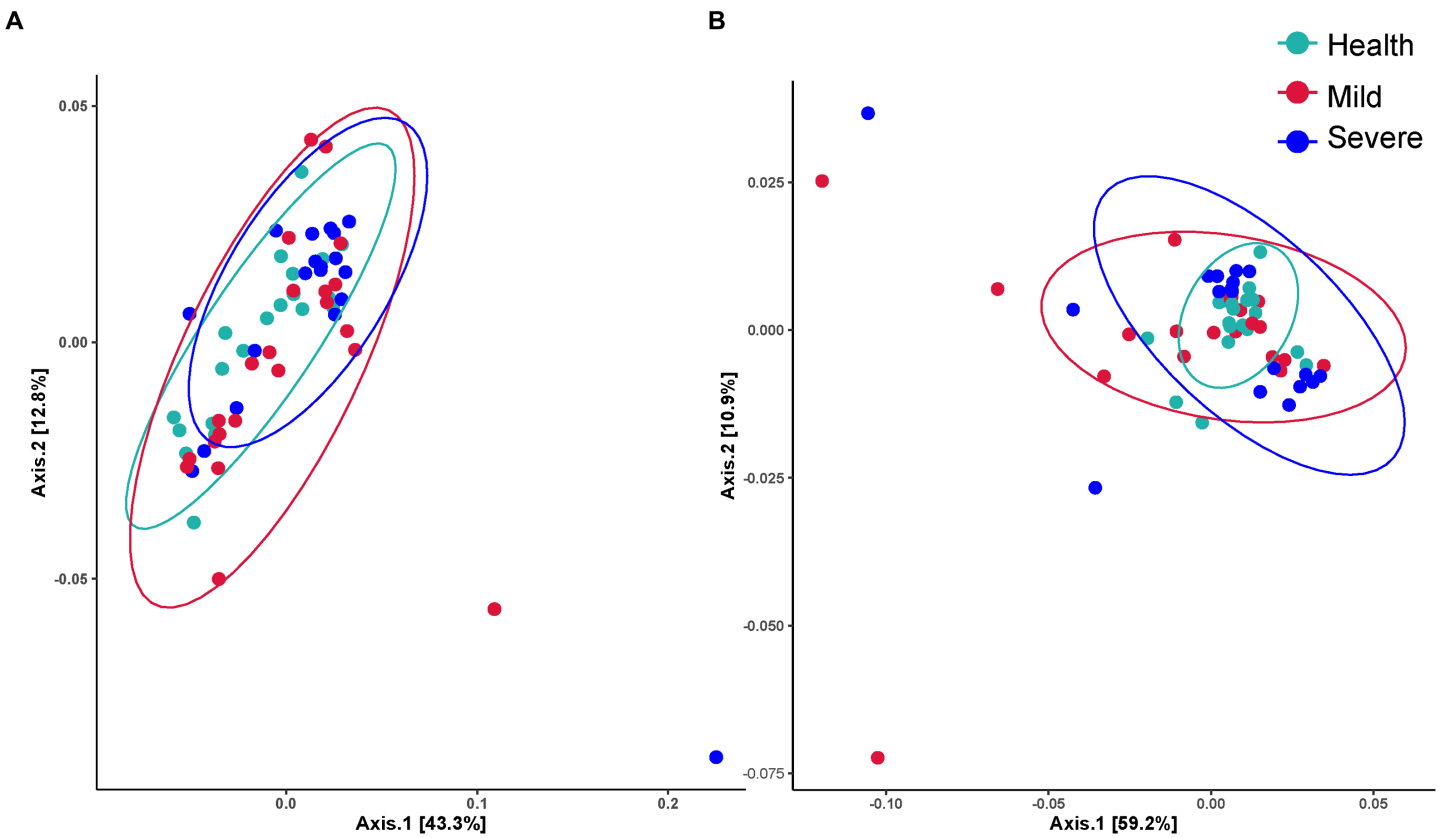
The beta diversity between the health, mild acne, and severe acne groups. **(A)** Bacteria flora. **(B)** Fungal flora.

The Shannon value was selected to show the difference in the alpha diversity between groups (Table 2). The *value of p*s obtained by the data statistics were all greater than 0.05, which indicated that there was no difference in the alpha diversity between different groups and sampling sites. Figure 1 shows the beta diversity between groups. For bacterial flora, it was obviously indicated that the beta diversity in the acne group has no difference with the health control. Besides, no significant difference between the mild and severe acne groups could be observed (Figure 1A). Contrastly, almost half of the samples in the mild and severe acne groups had significant differences with the healthy control group in the beta diversity of fungal flora (Figure 1B). For samples from different sampling sites, there was no difference between groups in the beta diversity of bacterial flora, while slight differences were observed between the severe group and the health group in the beta diversity of fungal flora within samples from the forehead, cheek, and chin (Supplementary Figures S2–S5).

**Table 2:** The Shannon value of different groups and sampling sites.

Sequencing methods	Sampling sites	Health	Mild	Serve	*p*-value
16S rRNA	Forehead	3.63 ± 1.56	3.96 ± 1.15	3.16 ± 0.64	0.458
	Cheek	3.81 ± 1.02	3.26 ± 1.60	3.81 ± 1.15	0.397
	Chin	3.83 ± 1.44	3.50 ± 1.07	2.80 ± 2.25	0.586
	Chest and back	3.54 ± 1.03	3.66 ± 1.44	3.65 ± 0.63	0.995
	Total	3.73 ± 1.21	3.66 ± 1.22	3.45 ± 1.15	0.816
18S rRNA	Forehead	1.53 ± 0.95	2.03 ± 1.35	1.33 ± 0.22	0.765
	Cheek	1.49 ± 0.76	2.19 ± 0.39	2.34 ± 1.11	0.251
	Chin	1.49 ± 0.69	1.18 ± 0.41	1.47 ± 0.27	0.533
	Chest and back	1.43 ± 0.56	2.42 ± 1.41	1.55 ± 0.46	0.302
	Total	1.49 ± 0.71	1.94 ± 1.14	1.74 ± 0.78	0.321

### 3.3. The skin microbiota structure

**Figure 2:**
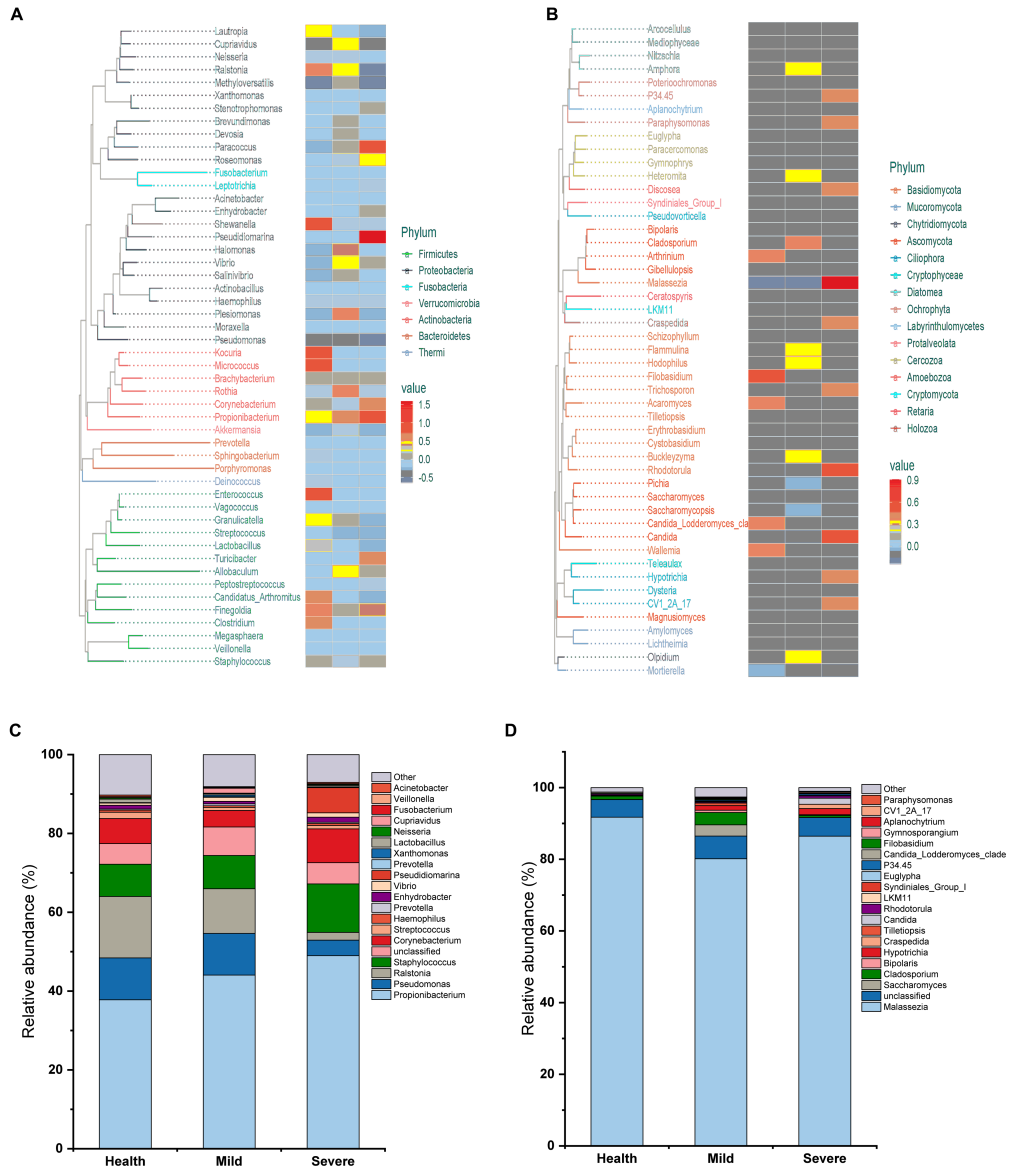
The composition of skin microbiota in different groups at the phylum and genus level. **(A)** Evolutionary tree for bacteria flora. **(B)** Evolutionary tree for fungal flora. **(C)** The top 20 genera in bacteria flora. **(D)** The top 20 genera in fungal flora.

The evolutionary trees were applied to exhibit the structure of skin microbiota (Figures 2A,B). At the phylum level, bacterial flora was mainly composed of *Firmicutes*, *Proteobacteria*, *Fusobacteria*, *Verrucomicrobia*, *Actinobacteria*, *Bacteroidetes*, and *Thermi*. Meanwhile, for fungal flora, the species were mainly derived from 15 phyla represented by *Basidomycota*. At the genus level, more than 50 genera were found both in the bacterial and fungal flora and the heatmap indicated that there are differences between groups in the relative abundance of some genera. To clearly show the composition of skin microbiota at the genus level, the top 20 genera in relative abundance values were displayed in a column chart, and the other genera were classified as others (Figures 2C,D). For bacterial flora, *Propionibacterium* has the highest abundance, then followed by *Pseudomonas*, *Ralstonia*, *Staphylococcus*, *Corynebacterium*, etc. Unsurprisingly, *Malassezia* occupied the highest abundance in the structure of fungal flora, which was more than 80% in all groups.

### 3.4. Significant differences at the genus level

**Figure 3:**
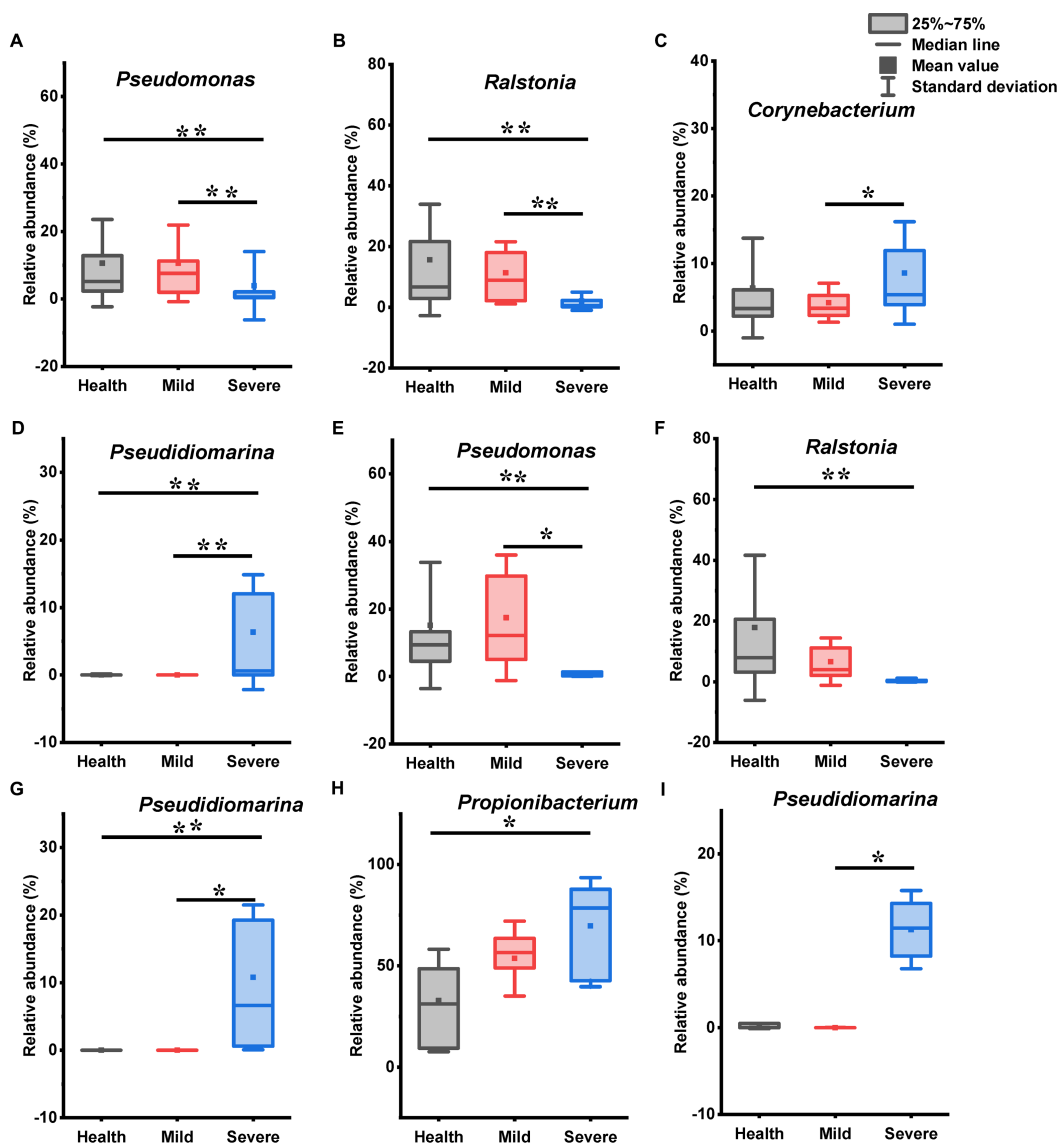
The genus with statistical difference between groups in the bacteria flora. **(A–D)** Samples from all sites; **(E–G)** samples from cheek; **(H)** samples from chin; **(I)** samples from chest and back. *0.01 < *p* < 0.05, significant difference; **p < 0.01, highly significant difference.

**Figure 4:**
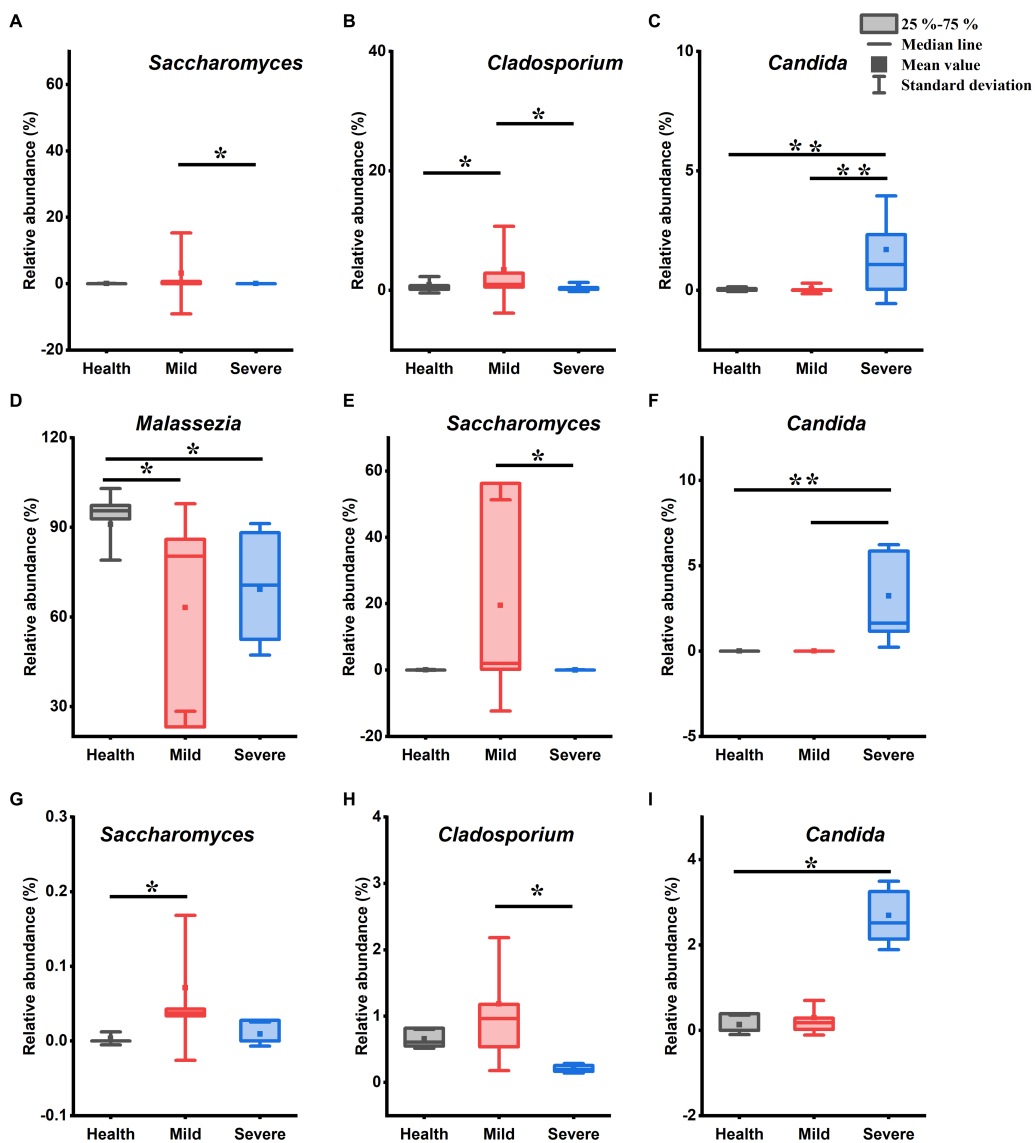
The genus with statistical difference between groups in the fungal flora. **(A–C)** Samples from all sites; **(D–F)** samples from cheek; **(G)** samples from chin; **(H,I)** samples from chest and back. *0.01 < *p* < 0.05, significant difference; **p < 0.01, highly significant difference.

To excavate the key species that lead to the development of acne lesions, the species with a relative abundance above 1% in bacterial flora and above 0.1% in fungal flora were selected to statistical analysis their differences between different groups (Figures 3, 4). At last, *Propionibacterium*, *Pseudomonas*, *Ralstonia*, *Staphylococcus*, and *Corynebacterium* were chosen in bacterial flora. Moreover, *Pseudidiomarina* was also selected due to a sharply increased could be observed in the severe group. For fungal flora, 6 genera were chosen including *Malassezia*, *Saccharomyces*, *Cladosporium*, *Hypotrichia*, *Craspedida*, and *Candida*.

For bacterial flora, there were significant differences between the health, mild, and severe groups in the relative abundance of *Pseudomonas*, *Ralstonia*, *Corynebacterium*, and *Pseudidiomarina* (Figures 3A–D). Among them, the relative abundance of *Pseudomonas* and *Ralstonia* was sharply reduced in the severe group when compared with that in the mild and health groups (Figures 3A,B, *p* < 0.01, Kruskal-Wallis test). Moreover, there was a difference in the relative abundance of *Corynebacterium* between the mild group and the severe group (Figure 3C, 0.01 < *p* < 0.05, Kruskal-Wallis test). Notably, *Pseudidiomarina* was not identified from many samples in the mild group and the health group. However, it existed with a high abundance in the severe group (Figure 3D). Interestingly, no difference was found between the mild group and the healthy group. For sampling sites, some genera abundance differences between groups were observed in the samples from the cheek, chin, and chest back (Figures 3E–I). Within the samples from the subjects’ cheek, there were 3 genera abundance had statistical differences between groups (Figures 3E–G). Compared with the health group, the relative abundance of *Pseudomonas* and *Ralstonia* reduced while the relative abundance of *Pseudidiomarina* increased in the severe group (*p* < 0.01, Kruskal-Wallis test). The mild group has differences from the severe group in the relative abundance of *Pseudomonas* and *Pseudidiomarina* (0.01 < *p* < 0.05, Kruskal-Wallis test). For the samples from the chin, only the abundance of *Propionibacterium* exhibited a difference between the health group and the severe group (Figure 3H, 0.01 < *p* < 0.05, LSD test), while for samples from the chest and back, the relative abundance of *Pseudidiomarina* in the mild group has a statistical difference from that in the severe group (Figure 3I, 0.01 < *p* < 0.05, Kruskal-Wallis test).

Figure 4 shows the genera that have a difference in the relative abundance between groups. Compared with the health group, both the mild group and the severe group has only one genus abundance slightly increased, they were *Cladosporium* (Figure 4B, 0.01 < *p* < 0.05, Kruskal-Wallis test) and *Candida* (Figure 4C, *p* < 0.01, Kruskal-Wallis test), respectively. In addition, three genera abundance had significant differences between the mild group and the severe group. Among them, in the mild group, the *Saccharomyces* (Figure 4A, 0.01 < *p* < 0.05, Kruskal-Wallis test) and *Cladosporium* (Figure 4B, *p* < 0.01, Kruskal-Wallis test) abundance increased while the abundance of *Candida* reduced (Figure 4C, *p* < 0.01, Kruskal-Wallis test). For the samples from the cheek, after acne occurred, there was a slightly reduced trend in the abundance of *Malassezia* (Figure 4D, 0.01 < *p* < 0.05, Kruskal-Wallis test), while the relative abundance of *Saccharomyces* and *Candida* separately increased in the mild group and the severe group (Figures 4E,F). The abundance of *Saccharomyces* increased in the samples of acne groups which was taken from the chin, however, only the mild group has a statistical difference from that in the healthy group (Figure 4G, 0.01 < *p* < 0.05, Kruskal-Wallis test). There were two genera abundance exhibited differences between groups for the samples from chest and back. Compared with the severe group, the *Cladosporium* abundance increased in the mild group (Figure 4H, 0.01 < *p* < 0.05, Kruskal-Wallis test) and the *Candida* abundance reduced in the health group (Figure 4I, 0.01 < *p* < 0.05, Kruskal-Wallis test).

### 3.5. Microbial network analysis

**Figure 5:**
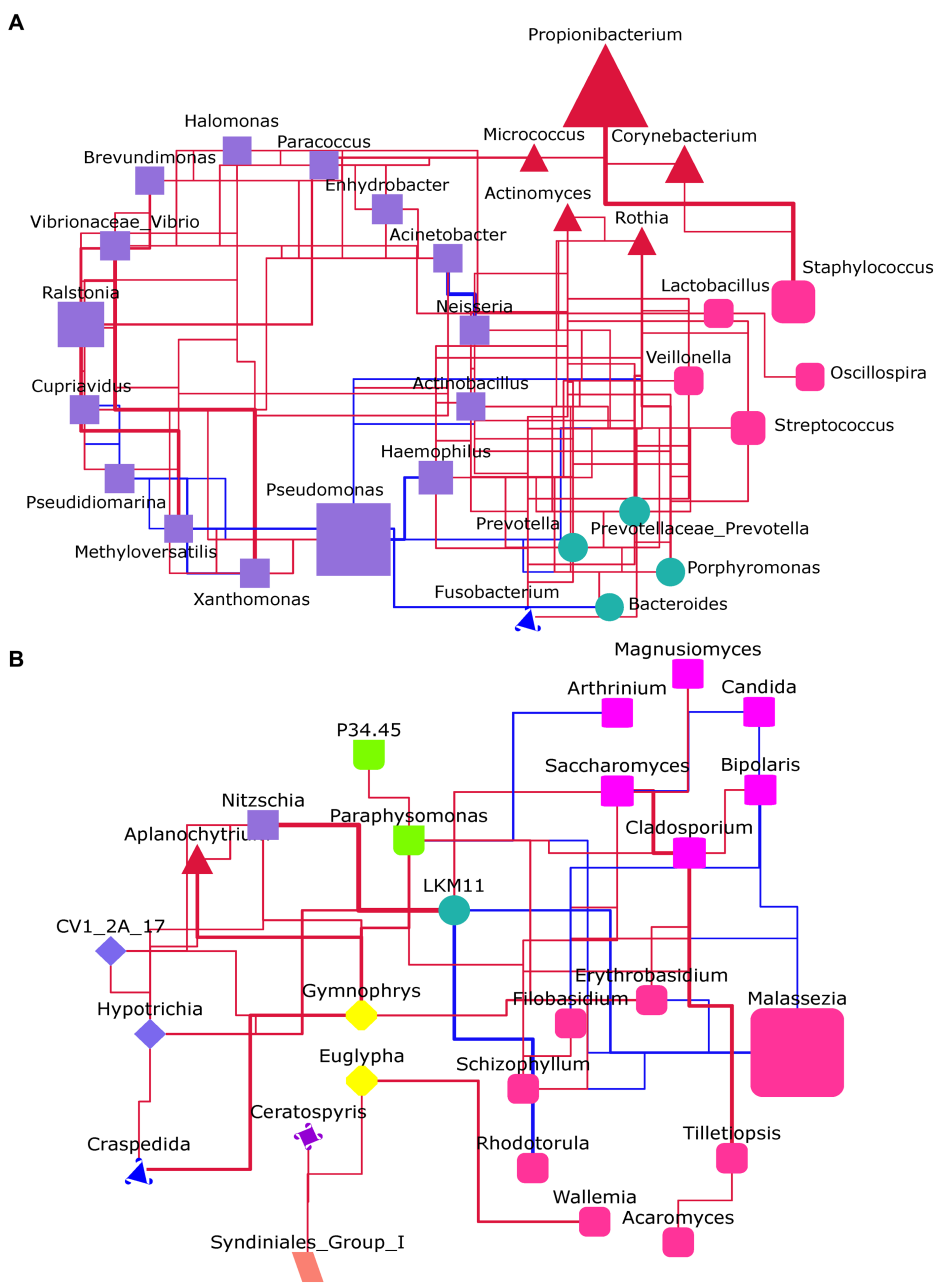
The microbial network analysis at the genus level. **(A)** For the bacteria flora. **(B)** For the fungal flora. The genera within the same phylum had the same shape and color. The size of the shape represents the abundance of genus. Red line: positive correlation; Blue line: negative correlation; The thicker the line, the stronger the correlation.

The interactions between microbial were presented by constructing a network based on the Spearman correlation between genera (Figure 5). It showed that most of the genera in the bacterial flora had a positive correlation with others. Especially, there was a close positive relationship between *Propionibacterium* and *Staphylococcus*. Notably, almost all genera connected with *Pseudomonas* had a negative correlation with it (Figure 5A). In the fungal flora, *Malassezia* has a negative relationship with all the connected genera (Figure 5B).

### 3.6. Functional prediction of the skin microbiota of acne patients

The KEGG database and the MetaCyc database were employed to predict the function of the bacterial flora and the fungal flora, respectively.

**Figure 6:**
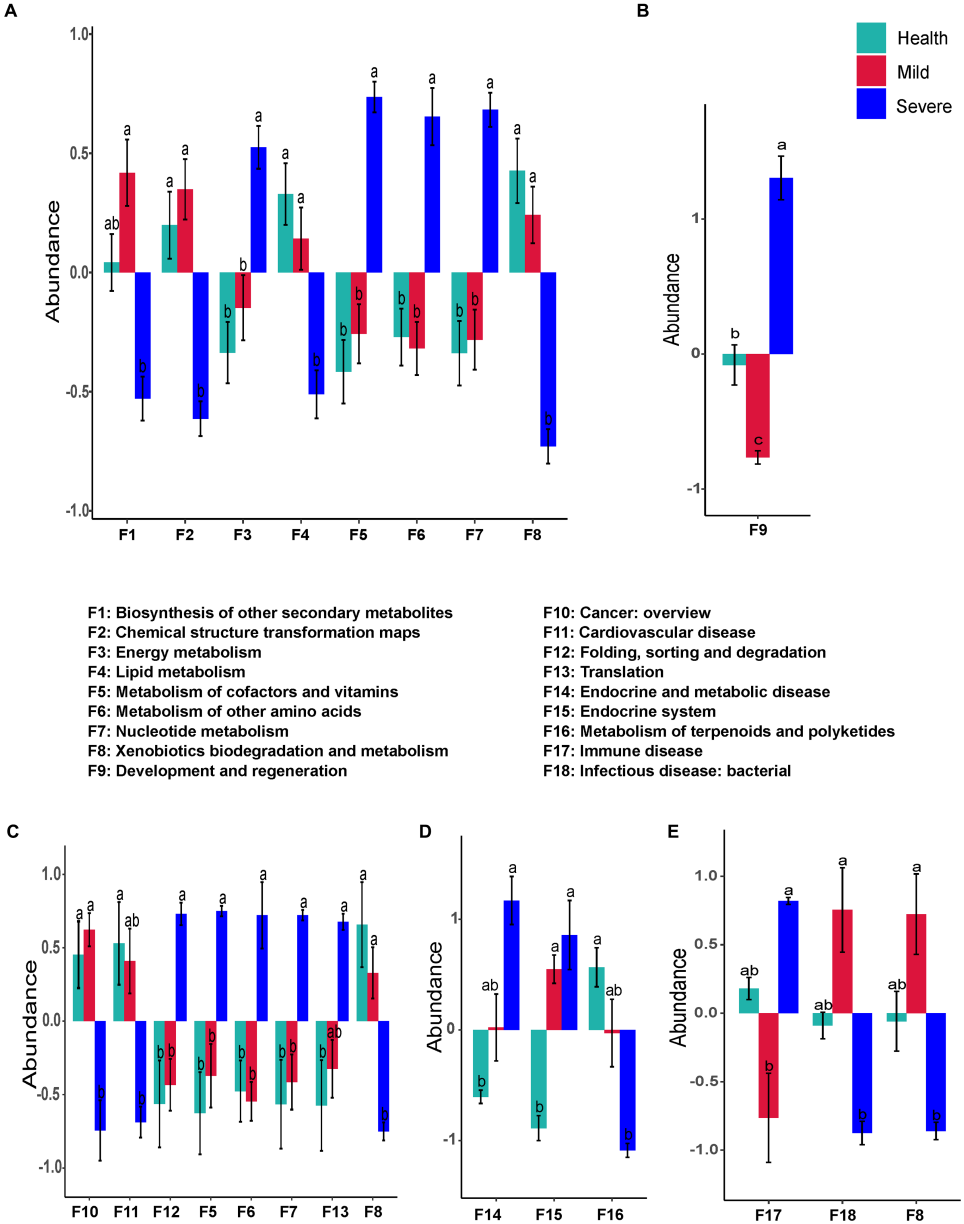
The bacteria flora predicted functions that had a significant difference between groups. **(A)** Total samples from all sampling sites; **(B)** samples from the forehead; **(C)** samples from the cheek; **(D)** samples from the chin; **(E)** samples from check and back. The different alphabet on the column represented there was a significant difference between groups.

Figure 6 shows the bacterial flora predicted functions that have a significant difference between groups. There was no difference in the abundance of all functions between the health group and the mild group. Nevertheless, the abundance of eight predicted functions that have significant differences between the severe group and the health group were observed. Among them, the abundance of four predicted functions decreased, they were biosynthesis of other secondary metabolites, chemical structure transformation maps, lipid metabolism, and xenobiotics biodegradation and metabolism (F1, F2, F4, and F8, respectively, Figure 6A). The four abundance-increased predicted functions in the severe group were energy metabolism, metabolism of cofactors and vitamins, metabolism of other amino acids, and nucleotide metabolism (F3, F5, F6, and F7, respectively, Figure 6A).

For the sampling sites, only one function (development and regeneration) was found that has a difference between groups in the samples from the forehead (Figure 6B). The functional prediction of samples from the cheek indicated that the abundance of eight predicted functions were altered in the severe group. Among them, five abundance-increased predicted functions including metabolism of cofactors and vitamins, metabolism of other amino acids, nucleotide metabolism, folding, sorting and degradation, and translation (F5, F6, F7, F12, and F13, respectively), while three abundance-decreased predicted functions were xenobiotics biodegradation and metabolism, cancer:overview, and cardiovascular disease (F8, F10, and F11, respectively, Figure 6C). Compared with the health group, the abundance of three predicted functions in the samples from the chin of the severe group was altered. The abundance-decreased predicted function was metabolism of terpenoids and polyketides (F16), while the other two abundance-increased predicted functions were endocrine and metabolic disease and endocrine system (F14, F15, Figure 6D). Surprisingly, there was no difference between the acne groups and the health group in the samples from the chest and back, but the abundance of three predicted functions has a significant difference between the mild group and the severe group (Figure 6E). Compared with the mild group, the abundance of immune disease (F17) sharply increased in the severe group, while the abundance of xenobiotics biodegradation and metabolism (F8) and infectious disease: bacterial (F18) markedly decreased.

**Figure 7:**
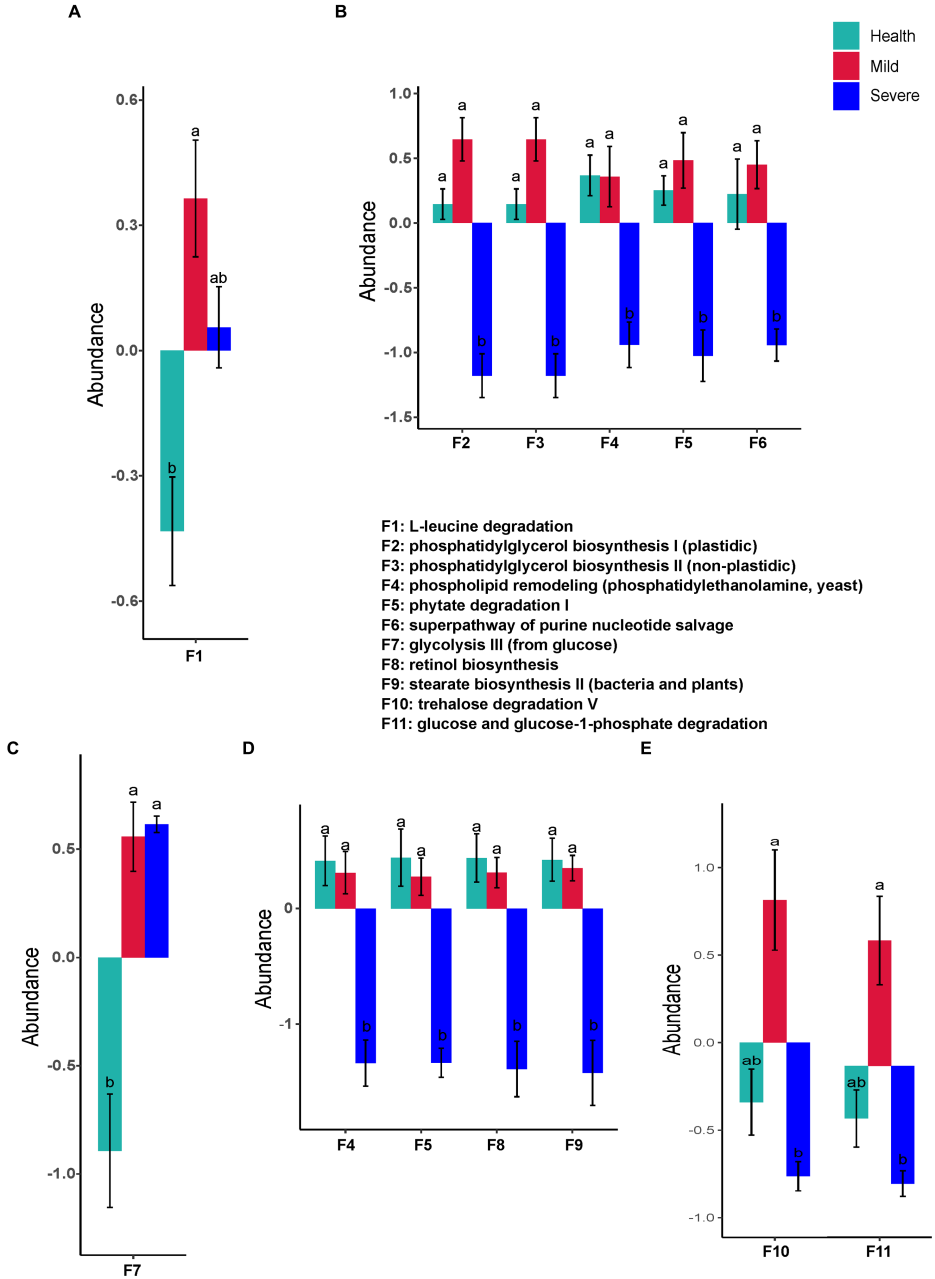
The fungal flora predicted functions that had a significant difference between groups. **(A)** Total samples from all sampling sites; **(B)** samples from the forehead; **(C)** samples from the cheek; **(D)** samples from the chin; **(E)** samples from check and back. The different alphabet on the column represented there was a significant difference between groups.

Interestingly, only one predicted function (L-leucine degradation) of the fungal flora was found with a difference abundance between the groups. The abundance of L-leucine degradation in the mild group has a significant increase when compared with that in the health group (Figure 7A). Contrastly, there were significant differences in functional prediction of fungal flora in the samples from different sampling sites (Figures 7B–E). The marked alterations were observed in the samples from the forehead and the chin. A total of five and four changed functions were separately found in the severe group, moreover, the abundance of these predicted functions all decreased (Figures 7B,D). These abundance-decreased functions in the severe group were phosphatidylglycerol biosynthesis I (plastidic), phosphatidylglycerol biosynthesis II (non-plastidic), phospholipid remodeling (phosphatidylethanolamine, yeast), phytate degradation I, superpathway of purine nucleotide salvage, retinol biosynthesis, and stearate biosynthesis II (bacteria and plants) (F2, F3, F4, F5, F6, F8, and F9, respectively). For the samples from the cheek, the abundance of glycolysis III (from glucose) significantly increased in the acne group when compared with that of the health group (Figure 7C). Similar to the results of functional prediction of bacteria flora in the samples from the chest and back, significant differences in the predicted functions of fungal flora were only found between the mild group and the severe group (Figure 7E). The abundance of trehalose degradation V (F10) and glucose and glucose-1-phosphate degradation (F11) was observed significantly decreased in the severe group.

## 4. Discussion

The colonization of human skin microorganisms depends on a variety of internal and external factors, such as host genetics, skin location, sex, age, ethnicity, geography, climate, environment, and lifestyle (Bay and Ring, 2022). To avoid external factors as much as possible, in this study, college students were recruited as subjects due to they are acne-prone people and they all live in school. Moreover, they have not been out of school during the sampling time of this study due to the COVID-19 epidemic prevention and control. Thus, the skin microbiota structure of these subjects was relatively stable.

For the difference in skin microbial diversity between acne patients and healthy people, there are currently reports of completely opposite results. There were two reports investigating Chinese acne patients’ skin microbiota suggested that the alpha diversity of skin microbiota in the acne group is greater than that in the healthy control group, while beta diversity also showed differences (Li et al., 2019; Shi et al., 2021). However, there were reports found that the diversity of skin microbiota in the acne group is no different from that in the healthy group, and even reports suggested that the diversity of skin microbiota in acne patients is significantly reduced (Cavallo et al., 2022). Recently, a report from Korea revealed that there was no difference in the diversity between acne and healthy group (Kim et al., 2021), which was consistent with this study that only a slight difference was found in the beta diversity of fungal flora between the different grade acne group and the health group (Table 2; Figure 2; Supplementary Figures S1–S4). From the literature analysis, we inferred that the key reason for obtaining different results might be due to the different sources of subjects. For example, in the two published articles from China institutions, the subjects were recruited from acne patients who came to the hospital. Compared with this study, the subjects’ different backgrounds such as jobs and lifestyles might significantly increase the diversity of the samples. In addition, different acne classification criteria, and differences in sampling environments and operations might also influence the obtained results. Therefore, it suggests we do better record in detail the various factors that may affect the skin microbiota during the implementation process. It will provide subsequent researchers with as many references as possible.

It has been for a long time recognized that one of the important acne pathogeneses is the overgrowth of some species of *Propionibacterium* and *Malassezia* (Fitz-Gibbon et al., 2013; Numata et al., 2014). *Propionibacterium acnes*, which is naming changed to *Cutibacterium acnes* (*C. acnes*), is the pathogenic bacteria most closely associated with acne (Achermann et al., 2014). It can promote sebum secretion, stimulate excessive keratinization of keratinocytes, aggravate inflammation, and lead to the formation of acne, papules, and pustules (Xu and Li, 2019). However, recently, there were studies indicated that *C. acnes* proliferation is not the cause of acne, and ecological imbalances between skin microbiota and different *C. acnes* types may play a more critical role in the development of acne (Barnard et al., 2016; Szabo et al., 2017). In addition, it has been reported that the relative abundance of *C. acnes* in the skin of patients with acne is comparable to that of healthy people, or slightly lower than that of healthy people (Barnard et al., 2016; Dreno et al., 2018). In this study, although the relative abundance of *Propionibacterium* increased after the acne occurred and an uptrend of abundance was observed with the acne grading increase, the statistical analysis indicated that there was no difference between groups (Figure 2C). Moreover, the genera like *Propionibacterium, Staphylococcus, Corynebacterium*, and *Malassezia*, which have a relatively high abundance in the skin microbiota and were reported as the most acne-associated microbes, were no obvious differences between groups in this study (Figures 3, 4). On the contrary, the abundance of some less reported Gram-negative bacteria and fungi such as *Pseudomonas*, *Ralstonia*, *Pseudidiomarina,* and *Candida* has a significant alteration in the severe group. Among them, the abundance of *Pseudomonas* and *Ralstonia* sharply reduced while that of *Pseudidiomarina* and *Candida* remarkably raised.

*Pseudomonas* is a kind of opportunistic pathogen and normally inhabits water-rich environments such as swimming pools and hot tubs (Morss-Walton et al., 2022). In the habitat that co-exists with *C. acnes*, there is a competitive relationship between them. The decline in *C. acnes* abundance has been reported often accompanied by an increase in its abundance (Chien et al., 2019; Morss-Walton et al., 2022). Consistent with these reports, the relative abundance of *Pseudomonas* was sharply reduced in the severe group when compared with that in the health group and the mild group (Figure 3A). Through network analysis, further verified the competitive relationship between them (Figure 5A). *Pseudomonas* has a negative correlation with all genera which it is connected, while *Propionibacterium*, on the contrary, has a positive correlation with the genus which it is connected. However, it should be noted that there is no direct correlation between them and their relationship deserved to be further investigated. *Ralstonia* and *Pseudomonas* belong to the phylum *Proteobacteria*. Similar to *Pseudomonas*, it usually colonizes in moist environments such as water and soil. The species from this genus are also opportunistic pathogens that could cause infections in human beings, especially in immunocompromised groups (Ryan and Adley, 2014). However, it may play a beneficial role in treating acne. It has been reported that *Ralstonia* has a close relationship with the metabolism of diglyceride and triglyceride in the skin of acne rats, the abundance of it increased after treating acne rats with cryptotanshinone (Zhu et al., 2022). *Pseudidiomarina* is a genus that has normally been found in the marine environment (Li et al., 2020). Until now, to the best of our knowledge, there is no report about its effects on skin disease. In this study, its abundance sharply increased in some samples from the severe group (Figure 3D), the existence of it in skin microbiota should be further confirmed. *Candida* is reported as an acne-causing bacterium due to its species together with *C. acnes* and *Staphylococcus aureus* could form a biofilm (Lee et al., 2022). The forming biofilm not only could against the traditional antimicrobial agents and host immune systems, but also provide inhabit spaces for microorganisms. Although its abundance is largely lower than that of *Malassezia*, results in this study suggested that it might be another targeted fungus except for *Malassezia* when treating acne (Figure 4). The skin microorganism that inhabits the surface of the skin includes both long-term and short-term survival. The long-term survival species normally belong to *Propionibacterium*, *Staphylococcus*, *and Malassezia*, while the short-term survival species are usually coming from Gram-negative bacteria, *Streptococcus*, and *Candida*. Previous studies have often focused on changes in the abundance of long-term survival species and their role in the development of acne. Currently, with the fast development of metagenomic technology, it is suggested that the vital role of the species with a low abundance in maintaining skin homeostasis should not be neglected (Leung et al., 2015). Thus, the role of *Pseudomonas*, *Ralstonia*, *Pseudidiomarina,* and *Candida* in acne pathogenesis deserved to pay more attention.

Body site is one of the important factors influencing the composition of skin microbiota, its impact is even higher than ethnicity in some reports (Perez Perez et al., 2016). Although there were reports that obtain samples from different acne sites, most of them just paid attention to facial acne or lack detailed reports because of too less samples for each site (Kim et al., 2021; Shi et al., 2021; Akaza et al., 2022). Thus, it is deserved to systematically investigate the structural characteristics of the skin microbiota in different acne areas and to excavate the biomarkers of sampling sites. In this study, in addition to dividing the face area into three sampling sites (including the forehead, cheek, and chin), we also sampled the torso area (including the chest and back) which has a low prevalence (1–14%) and is frequently underdiagnosed (Tan et al., 2022). Moreover, at least three samples were obtained in each sampling site of different groups. Finally, results proved that different acne sampling sites have different numbers and types of biomarkers. Among the four sampling sites, the cheek has the most biomarkers including *Pseudomonas*, *Ralstonia*, *Pseudidiomarina*, *Malassezia*, *Saccharomyces*, and *Candida*, while no biomarker was observed for the forehead (Figures 3E–G, 4D–F).

The alteration of skin microbiota composition bring about changes in the function of skin microbiota, which will inevitably affect the occurrence and development of skin diseases. In this study, there were significant differences at the abundance of several predicted functions between the severe group and the health group (Figures 6, 7). Especially, it should be noted that most of these functions are related to the metabolism of key substances such as lipid, cofactors and vitamins, and amino acids (Figure 6A). The alteration of these substances might significantly disturb host skin homeostasis by stimulating the keratinocytes and immune cells of the skin (Chen et al., 2018). Compared with the health group, the mild group almost has no difference from it in microbial composition and predicted functions (Figures 3–7). These results suggested that microbial might not play an important role in the pathogenesis of mild acne. It has been reported that acne is mediated by immunity which is a communicator of the gut-skin axis, while gut homeostasis could be affected by many factors such as diet, lifestyle, etc. (Sanchez-Pellicer et al., 2022; Yang et al., 2022). Combining this view with our results, for patients with mild acne, it is recommended to first adjust their diet and lifestyle or take probiotics to improve their immunity.

In this study, the skin composition of patients with mild and severe acne was investigated and compare with that of the health group. Our results suggested that the less reported Gram-negative bacteria (*Pseudomonas*, *Ralstonia*, and *Pseudidiomarina*) and *Candida* might play a vital role in the development of acne. This study provided new insight into the treatment of acne. However, there are limitations to this study. First, the number of participants in the study was small, especially the patients with severe acne. Second, limited by the disadvantages of 16S rRNA and 18S rRNA gene sequencing, the biomarkers at the species level were not been identified. At last, the correlation analysis between skin microbiota and their metabolisms with the clinical phenotype of acne was less. In the future, on the one hand, it is necessary to expand the number of cohorts and increase the collection of samples; On the other hand, the metagenomic should be combined with metabolomics to excavate the key biomarker and provide a theoretical basis for precise and personalized acne microbial therapy.

## Data availability statement

The datasets presented in this study can be found in online repositories. The names of the repository/repositories and accession number(s) can be found at: https://www.ncbi.nlm.nih.gov/bioproject/PRJNA949583/.

## Ethics statement

The studies involving human participants were reviewed and approved by the Ethics Committee of the Affiliated Hospital of Jiangnan University. The patients/participants provided their written informed consent to participate in this study.

## Author contributions

ZG: methodology, investigation, data curation, writing—original draft, project administration. YY, QW, and ML: methodology, investigation, data curation. LeZ: writing—review and editing. LiZ, and DD: conceptualization, writing—review and editing, and supervision. All authors contributed to the discussion and review of the manuscript.

## Funding

This work was supported by the Top Talent Support Program for Young and Middle-Aged People of Wuxi Health Committee (BJ2020060), the Wuxi Science and Technology Plan Project (Y20212016), and the National Key Research and Developmental Program of China (2021YFC2100300).

## Conflict of interest

The authors declare that the research was conducted in the absence of any commercial or financial relationships that could be construed as a potential conflict of interest.

## Publisher’s note

All claims expressed in this article are solely those of the authors and do not necessarily represent those of their affiliated organizations, or those of the publisher, the editors and the reviewers. Any product that may be evaluated in this article, or claim that may be made by its manufacturer, is not guaranteed or endorsed by the publisher.

## Supplementary material

SUPPLEMENTARY FIGURE S1 The illustration of sampling collection.

SUPPLEMENTARY FIGURE S2 The beta diversity of skin microbiota of samples from the forehead.

SUPPLEMENTARY FIGURE S3 The beta diversity of skin microbiota of samples from the cheek.

SUPPLEMENTARY FIGURE S4 The beta diversity of skin microbiota of samples from the chin.

SUPPLEMENTARY FIGURE S5 The beta diversity of skin microbiota of samples from the chest and back.
