# Supplementary figures and images for: New insights into the characteristic skin microorganisms in different grades of acne and different acne sites
Source: Front Microbiol. 2023 Apr 27;14:1167923. doi: 10.3389/fmicb.2023.1167923 (PMC10172595; doi:10.3389/fmicb.2023.1167923)

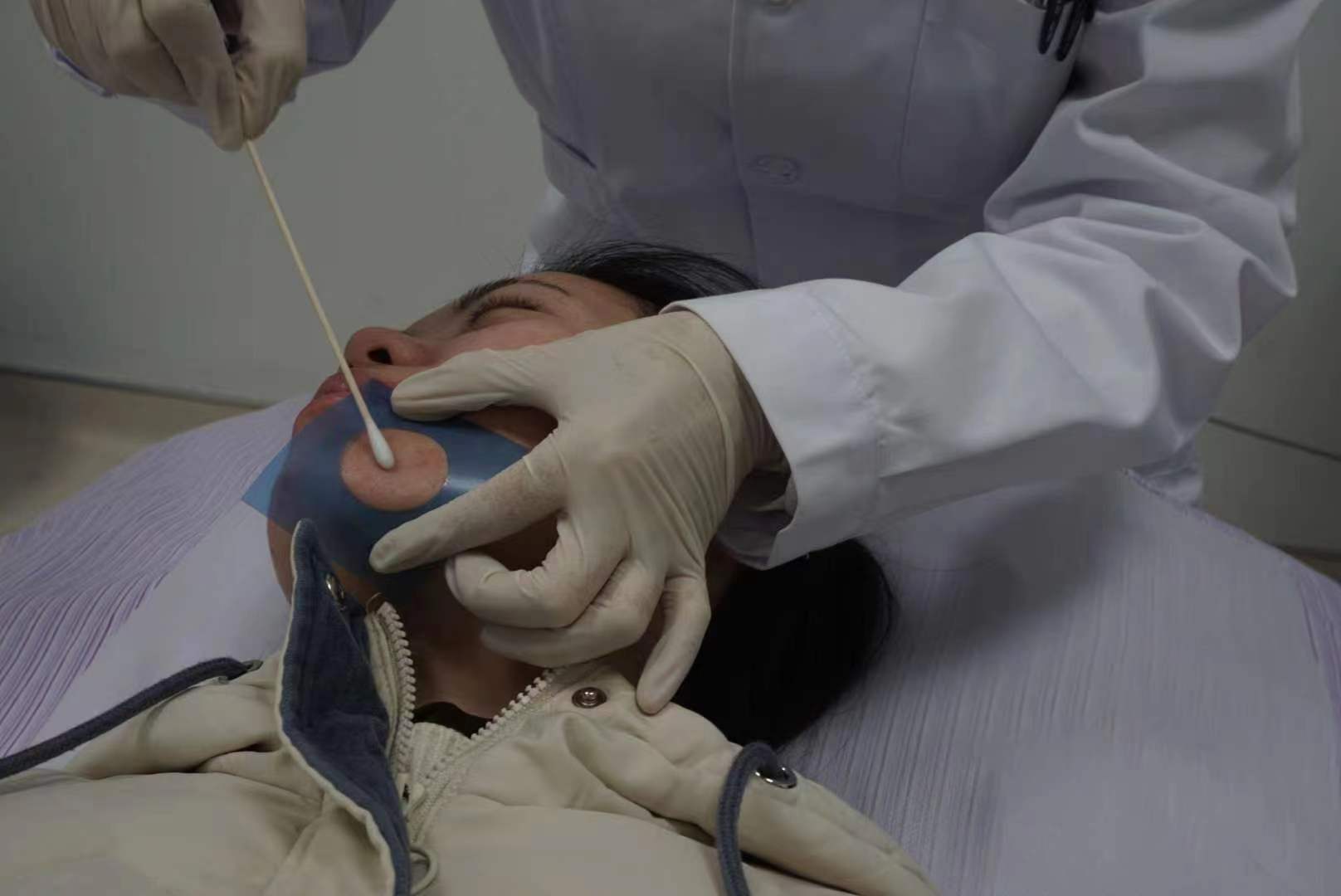

Supplement: SUPPLEMENTARY FIGURE S1 — The illustration of sampling collection. [file Image_1.jpg]

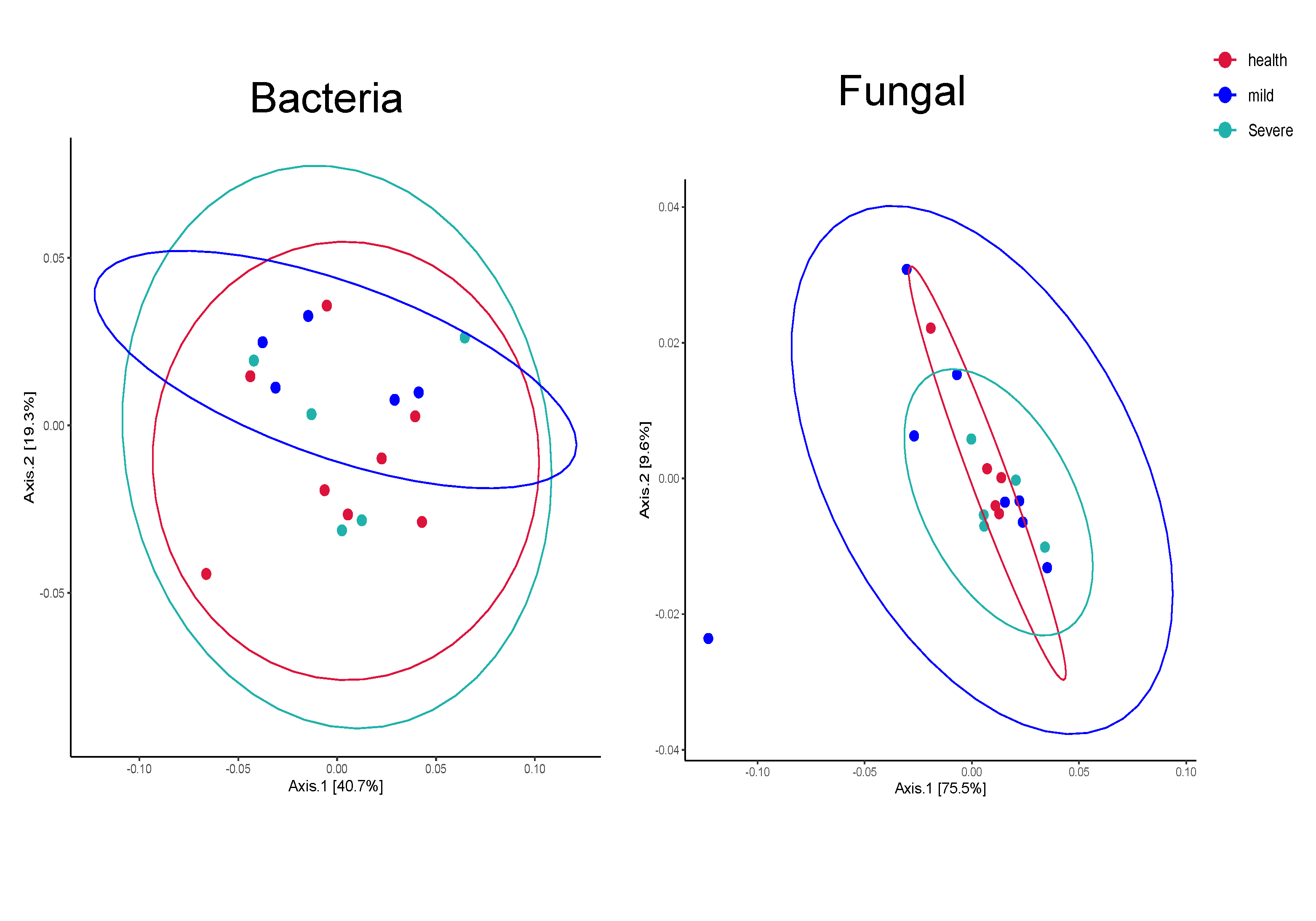

Supplement: SUPPLEMENTARY FIGURE S2 — The beta diversity of skin microbiota of samples from the forehead. [file Image_2.tif]

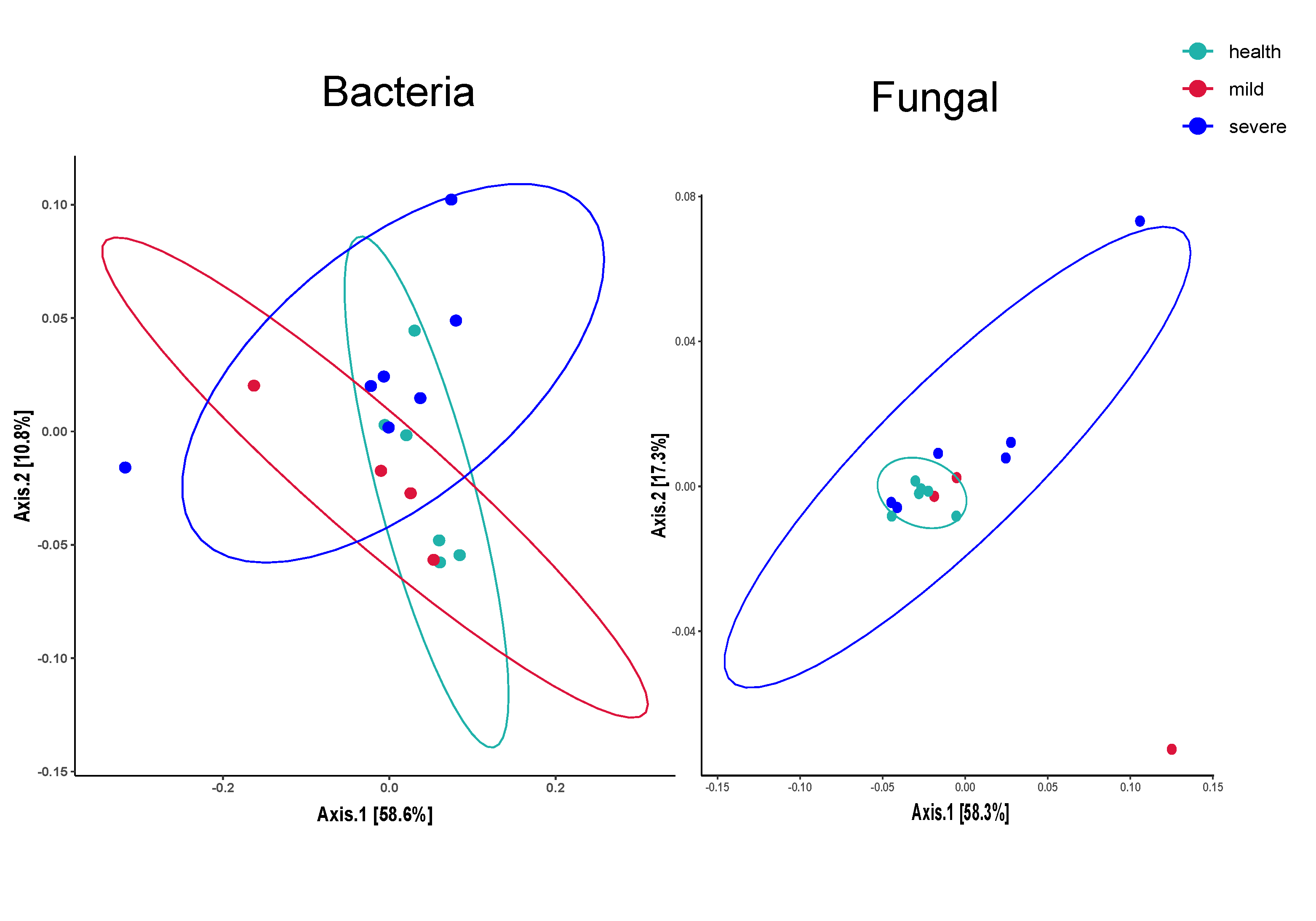

Supplement: SUPPLEMENTARY FIGURE S3 — The beta diversity of skin microbiota of samples from the cheek. [file Image_3.tif]

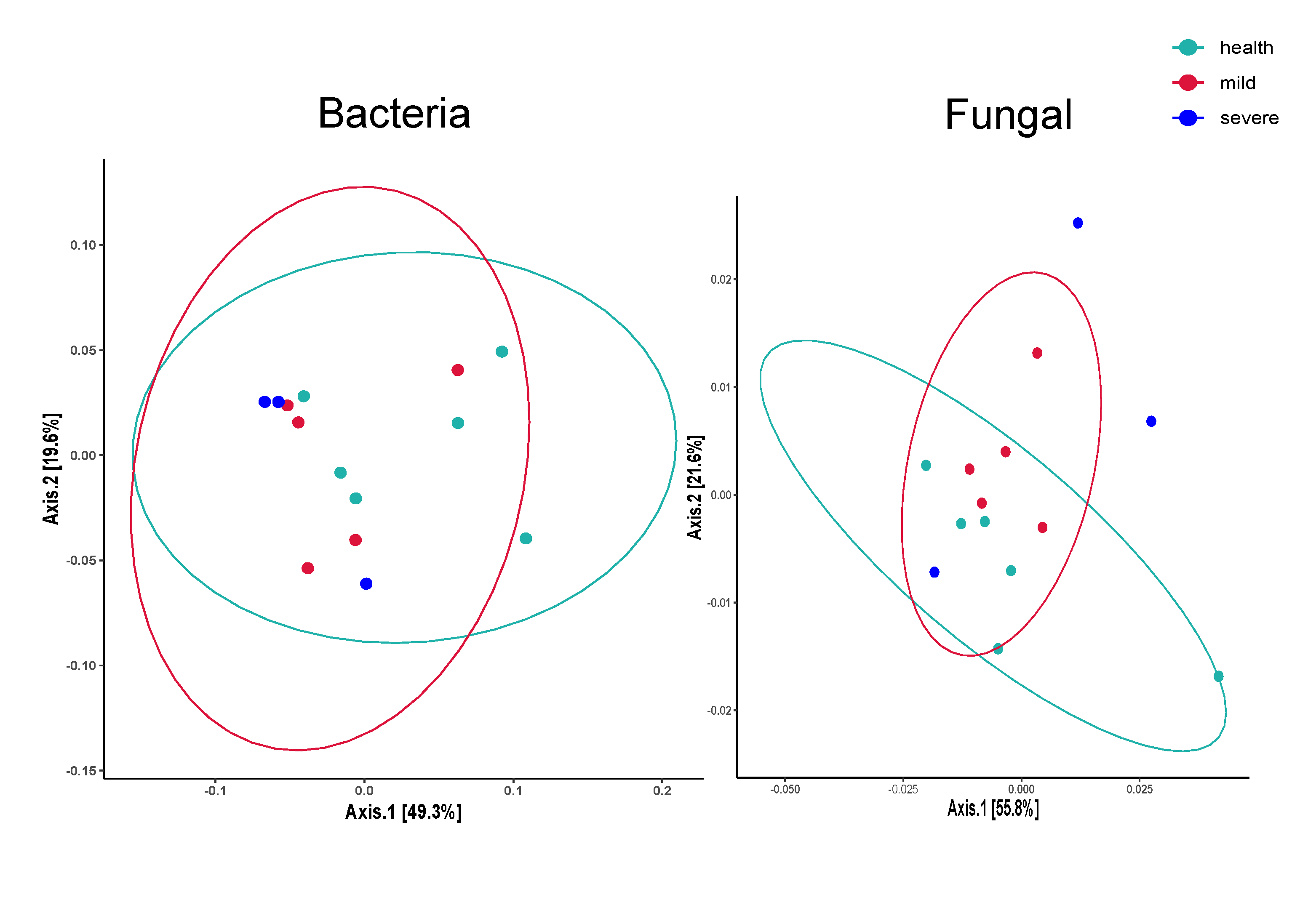

Supplement: SUPPLEMENTARY FIGURE S4 — The beta diversity of skin microbiota of samples from the chin. [file Image_4.tif]

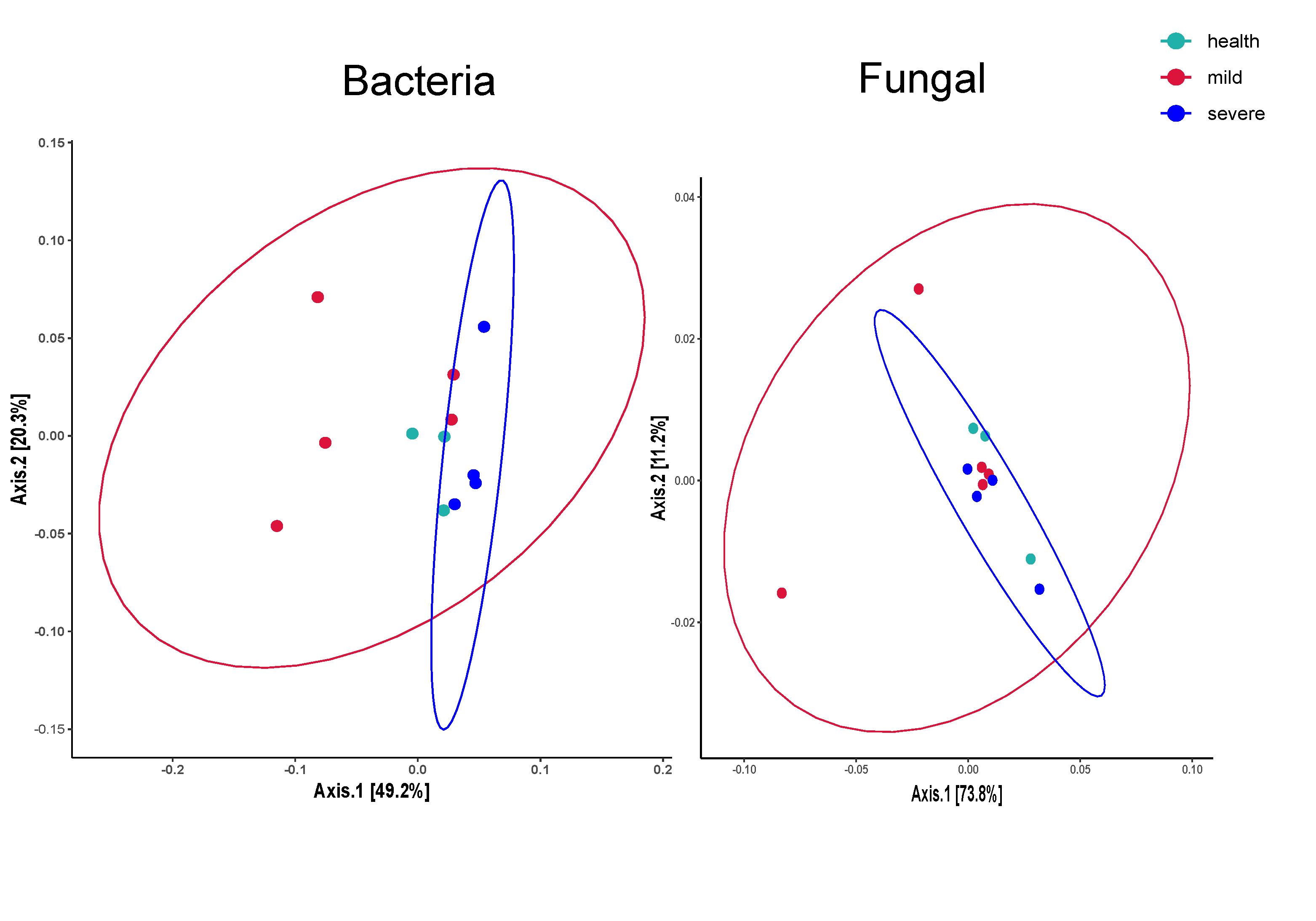

Supplement: SUPPLEMENTARY FIGURE S5 — The beta diversity of skin microbiota of samples from the chest and back. [file Image_5.tif]
